# Supplementary material for: Intensive sea urchin harvest rescales Paracentrotus lividus population structure and threatens self-sustenance
Source: PeerJ. 2023 Nov 20;11:e16220. doi: 10.7717/peerj.16220 (PMC10666612; doi:10.7717/peerj.16220)
Supplement: Supplemental Information 1 [file peerj-11-16220-s001.docx]

| **Month** | **Period 1** | **Period 2** | **Period 3** | **Period 4** | **Period 5** | **Period 5 (protected)** |
| --- | --- | --- | --- | --- | --- | --- |
| June | 19.7±0.3 | 21.9±0.3 | 20.7±0.2 | 22.9±0.2 | 21.7±0.2 | 21.7±0.2 |
| July | 23.5±0.3 | 25.4±0.1 | 23.1±0.2 | 24.0±0.1 | 25.3±0.1 | 25.4±0.1 |
| Aug | 24.6±0.1 | 24.7±0.1 | 23.8±0.1 | 24.9±0.2 | 26.2±0.2 | 26.4±0.2 |
| Sep | 23.3±0.2 | 24.1±0.2 | 24.1±0.1 | 23.0±0.2 | 24.8±0.1 | 24.9±0.1 |
| Oct | 22.1±0.1 | 20.7±0.2 | 22.0±0.1 | 21.4±0.1 | 21.9±0.1 | 22.0±0.1 |
| Nov | 17.8±0.4 | 19.0±0.2 | 19.4±0.2 | 18.0±0.2 | 19.0±0.2 | 19.0±0.2 |
| Dec | 15.3±0.1 | 16.1±0.1 | 17.6±0.1 | 14.9±0.1 | 15.2±0.1 | 15.3±0.1 |
| Jan | 14.4±0.1 | 15.3±0.1 | 15.1±0.2 | 14.3±0.0 | 14.2±0.1 | 14.2±0.1 |
| Feb | 13.9±0.1 | 14.6±0.1 | 14.2±0.1 | 13.8±0.1 | 13.6±0.0 | 13.6±0.0 |
| Mar | 13.6±0.1 | 14.2±0.1 | 14.9±0.1 | 13.6±0.0 | 14.0±0.1 | 14.0±0.1 |
| Apr | 15.7±0.1 | 16.0±0.1 | 16.4±0.1 | 15.7±0.3 | 15.2±0.1 | 15.2±0.1 |
| May | 17.3±0.2 | 17.0±0.2 | 18.5±0.3 | 17.7±0.2 | 16.4±0.1 | 16.5±0.1 |
| Annual avg | 18.4±02 | 19.1±0.2 | 19.2±0.2 | 18.7±0.1 | 19±0.1 | 19±0.1 |
| Fall/Winter avg | 16.2±0.2 | 16.7±0.1 | 17.2±0.1 | 16±0.1 | 16.3±0.1 | 16.4±0.1 |
