# Supplementary material for: Intensive sea urchin harvest rescales Paracentrotus lividus population structure and threatens self-sustenance
Source: PeerJ. 2023 Nov 20;11:e16220. doi: 10.7717/peerj.16220 (PMC10666612; doi:10.7717/peerj.16220)
Supplement: Supplemental Information 2 [file peerj-11-16220-s002.docx]

| **Month** | **Period 1** | **Period 2** | **Period 3** | **Period 4** | **Period 5** |
| --- | --- | --- | --- | --- | --- |
| June | 1.08 ± 0.03 | 0.66 ± 0.02 | 0.84 ± 0.02 | 0.57 ± 0.02 | 0.72 ± 0.02 |
| July | 0.42 ± 0.01 | 0.68 ± 0.02 | 0.82 ± 0.03 | 0.91 ± 0.03 | 0.62 ± 0.02 |
| Aug | 0.68 ± 0.02 | 0.67 ± 0.02 | 0.74 ± 0.02 | 0.63 ± 0.02 | 0.61 ± 0.02 |
| Sep | 0.86 ± 0.03 | 0.84 ± 0.02 | 0.74 ± 0.03 | 1.05 ± 0.03 | 0.59 ± 0.02 |
| Oct | 0.72 ± 0.02 | 0.90 ± 0.03 | 0.68 ± 0.02 | 1.05 ± 0.04 | 1.01 ± 0.04 |
| Nov | 1.90 ± 0.05 | 1.32 ± 0.05 | 1.06 ± 0.04 | 1.57 ± 0.05 | 1.08 ± 0.04 |
| Dec | 1.07 ± 0.04 | 0.30 ± 0.01 | 0.61 ± 0.02 | 1.89 ± 0.04 | 1.39 ± 0.04 |
| Jan | 1.50 ± 0.05 | 1.53 ± 0.04 | 1.49 ± 0.04 | 1.59 ± 0.04 | 2.21 ± 0.05 |
| Feb | 1.47 ± 0.03 | 1.93 ± 0.04 | 1.16 ± 0.05 | 1.39 ± 0.03 | 1.12 ± 0.05 |
| Mar | 1.25 ± 0.04 | 1.36 ± 0.04 | 1.03 ± 0.04 | 1.78 ± 0.03 | 1.30 ± 0.04 |
| Apr | 0.99 ± 0.03 | 1.03 ± 0.04 | 0.89 ± 0.02 | 0.83 ± 0.03 | 1.01 ± 0.02 |
| May | 0.95 ± 0.03 | 1.13 ± 0.03 | 0.68 ± 0.02 | 0.81 ± 0.02 | 1.29 ± 0.04 |
| Annual avg | 1.07 ± 0.01 | 1.03 ± 0.01 | 0.89 ± 0.01 | 1.17 ± 0.01 | 1.08 ± 0.01 |
| Fall/winter avg | 1.31 ± 0.01 | 1.22 ± 0.01 | 1.00 ± 0.01 | 1.55 ± 0.01 | 1.36 ± 0.01 |
