# Supplementary material for: Intensive sea urchin harvest rescales Paracentrotus lividus population structure and threatens self-sustenance
Source: PeerJ. 2023 Nov 20;11:e16220. doi: 10.7717/peerj.16220 (PMC10666612; doi:10.7717/peerj.16220)
Supplement: Supplemental Information 3 [file peerj-11-16220-s003.docx]

| Size-class (mm) | Period 1 | Period 5 | Control (Period 5) |
| --- | --- | --- | --- |
| TD<10 | 0.4±0.2 | 0.1±0.1 | 0.1±0.1 |
| 10≤TD<20 | 1.3±0.3 | 2.5±1.2 | 0.3±0.2 |
| 20≤TD<30 | 2.1±0.4 | 1.1±0.4 | 0.4±0.2 |
| 30≤TD<40 | 3±0.3 | 1±0.2 | 0.5±0.3 |
| 40≤TD<50 | 2.7±0.3 | 1.2±0.4 | 0.8±0.4 |
| 50≤TD<60 | 0.6±0.2 | 0.5±0.1 | 0.7±0.4 |
| TD≥60 | 0 | 0 | 0.1±0.1 |
| Tot. pop. density | 10±1.7 | 6.7±2.4 | 3.8±2 |
|  |  |  |  |
