# Supplementary figures and images for: Intensive sea urchin harvest rescales Paracentrotus lividus population structure and threatens self-sustenance
Source: PeerJ. 2023 Nov 20;11:e16220. doi: 10.7717/peerj.16220 (PMC10666612; doi:10.7717/peerj.16220)

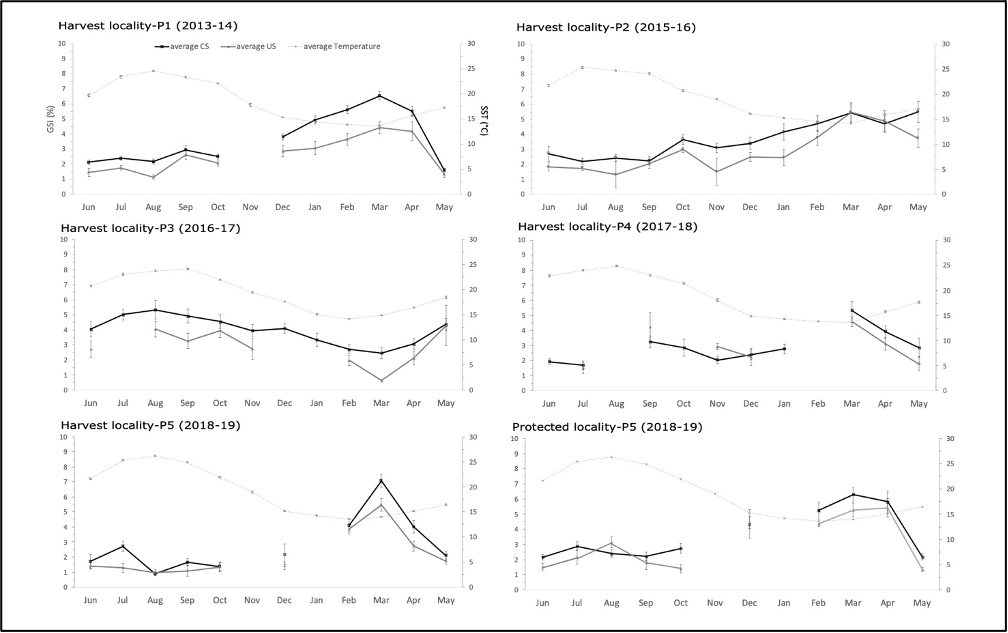

Supplement: Supplemental Information 4 — GSI is represented as mean ± standard deviation for CS size-class (black line) and US size-class (gray line). Due to adverse marine weather conditions, not all months were sampled. The reproductive cycle of 2014-15 was not estimated. Mean SST was also plotted (thinner gray line). [file peerj-11-16220-s004.png]

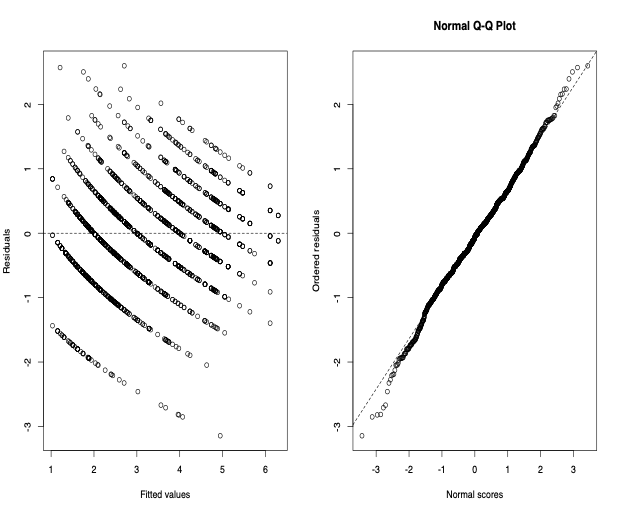

Supplement: Supplemental Information 5 [file peerj-11-16220-s005.png]

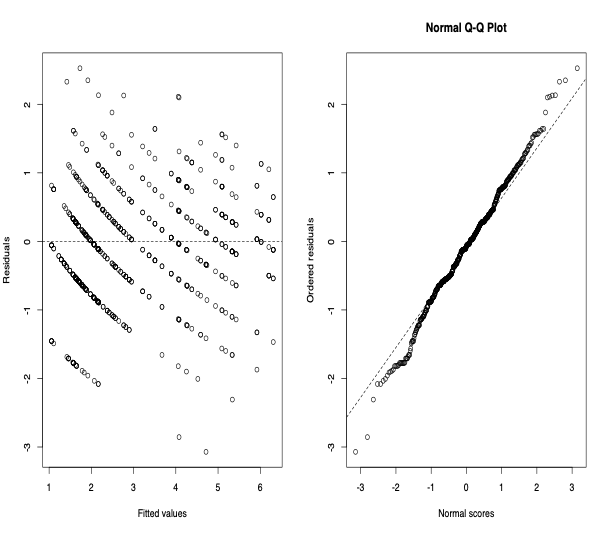

Supplement: Supplemental Information 6 [file peerj-11-16220-s006.png]

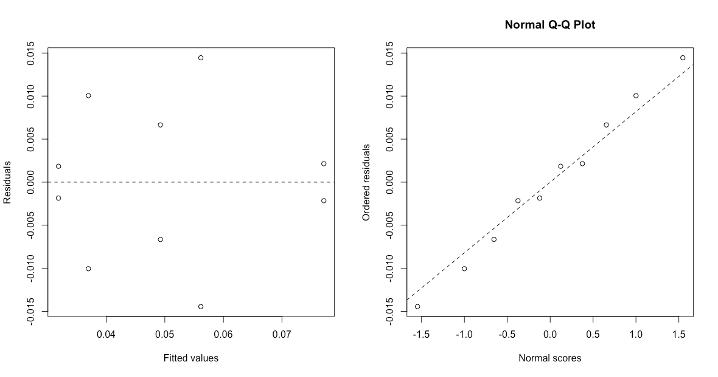

Supplement: Supplemental Information 7 [file peerj-11-16220-s007.png]
